# Ensemble graph neural network model for classification of major depressive disorder using whole-brain functional connectivity

**DOI:** 10.3389/fpsyt.2023.1125339

**Published:** 2023-03-23

**Authors:** Sujitha Venkatapathy, Mikhail Votinov, Lisa Wagels, Sangyun Kim, Munseob Lee, Ute Habel, In-Ho Ra, Han-Gue Jo

**Affiliations:** ^1^School of Computer Information and Communication Engineering, Kunsan National University, Gunsan, Republic of Korea; ^2^Department of Psychiatry, Psychotherapy and Psychosomatics, Medical Faculty, Uniklinik RWTH Aachen University, Aachen, Germany; ^3^Research Center Juelich, Institute of Neuroscience and Medicine: JARA-Institute Brain Structure Function Relationship (INM 10), Juelich, Republic of Korea; ^4^AI Convergence Research Section, Electronics and Telecommunications Research Institute, Gwangju, Republic of Korea

**Keywords:** major depressive disorder, deep learning, graph neural network, ensemble model, functional connectivity

## Abstract

Major depressive disorder (MDD) is characterized by impairments in mood and cognitive functioning, and it is a prominent source of global disability and stress. A functional magnetic resonance imaging (fMRI) can aid clinicians in their assessments of individuals for the identification of MDD. Herein, we employ a deep learning approach to the issue of MDD classification. Resting-state fMRI data from 821 individuals with MDD and 765 healthy controls (HCs) is employed for investigation. An ensemble model based on graph neural network (GNN) has been created with the goal of identifying patients with MDD among HCs as well as differentiation between first-episode and recurrent MDDs. The graph convolutional network (GCN), graph attention network (GAT), and GraphSAGE models serve as a base models for the ensemble model that was developed with individual whole-brain functional networks. The ensemble's performance is evaluated using upsampling and downsampling, along with 10-fold cross-validation. The ensemble model achieved an upsampling accuracy of 71.18% and a downsampling accuracy of 70.24% for MDD and HC classification. While comparing first-episode patients with recurrent patients, the upsampling accuracy is 77.78% and the downsampling accuracy is 71.96%. According to the findings of this study, the proposed GNN-based ensemble model achieves a higher level of accuracy and suggests that our model produces can assist healthcare professionals in identifying MDD.

## 1. Introduction

Depression is a major source of disability and disease burden worldwide, affecting about 264 million people. Major depressive disorder (MDD) is a serious psychological problem that can cause people to feel sad, lose interest, become listless, and have trouble thinking (1). Individuals who have suffered from MDD typically have difficulty adjusting together with their society. They have a low opinion of themselves, which ultimately leads to a decline in their performance at work. MDD can cause serious emotional problems and suicidal thoughts and behavior if it is not adequately recognized and treated (2). In patients suffering from MDD, abnormalities in large-scale brain connections have been identified more frequently in recent years. Depressed people revealed significantly disrupted connections between the task-related regions of the brain throughout a variety of task-directed functions, such as working memory, executive control, facial emotion perception, and impulse control (3, 4).

In recent years, research on MDD has focused on brain structure and function using morphological or neurobiological features. Functional magnetic resonance imaging (fMRI), magnetoencephalography (MEG), electroencephalography (EEG), and positron emission tomography (PET) are the common physiological methods employed in comparing people with MDD to healthy controls (HCs) (5). Researchers have found that patients with MDD have abnormal communication among the functional brain networks using functional connectivity (FC) of resting-state fMRI (R-fMRI), which detects synchronized and desynchronized spontaneous activity within anatomically diverse networks (6–8). In the present study, whole brain FC is extracted from R-fMRI data in order to determine whether or not the subject is MDD, and for classification between first-episode and recurrent MDD.

**Figure 1:**
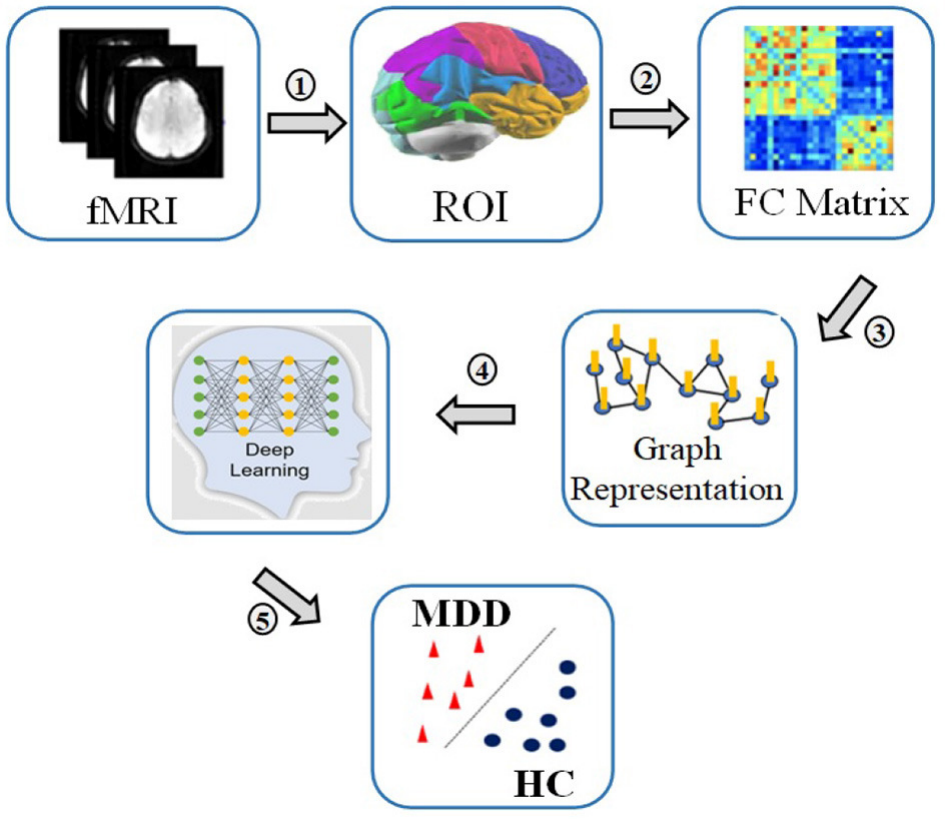
The process of MDD and HC classif ication: 1. Recording R-fMRI signal, 2. Extracting time series of regions of interest, 3. Calculating correlation matrices of the time series, 4. Converting correlations in graph representations, 5. Applying deep learning to classify between patients and controls.

Machine learning (ML) methods are of increasing interest for the medical industry at present, and it has emerged as an essential part of the diagnosis and treatment of conditions pertaining to oncology, neurology, and cardiology. The process involved in a typical deep learning pipeline for the identification of MDD can be highlighted as follows: region of interest (ROI) extraction from R-fMRI, functional connectivity matrix generation, graph construction, deep learning model training, and classification (9). Figure 1 depicts the processes required in identifying MDD from HCs.

This study presents a high-performance graph neural network (GNN)-based deep learning method for classifying individuals with MDD using R-fMRI data. In recent years, graph neural network (GNN) has become increasingly popular in graph-based learning. GNN become the optimal deep learning approach for analyzing graph-structured information. GNN algorithm combines node attributes, edge attributes, and graph topology by embedding node characteristics in a neural network and transferring data through the graph's edges. GNN can work well on non-euclidean domains and this is in contrast to traditional convolutional neural networks, which are limited to accepting only euclidean inputs (10). GNN has replaced older ML approaches due to their greater performance in analyzing graph-based information (11, 12).

Studies on MDD have progressed in recent years understanding changes of brain structure and function using morphological or neurobiological features. The studies summarized here used a number of machine learning algorithms, such as support vector machines (SVM), logistic regression, and neural networks, to differentiate between MDD and HCs using fMRI data. Especially, resting-state functional connectivity characteristics of the entire brain were studied in MDD. In order to distinguish individuals with MDD from controls, several studies (13–16) employed SVM-based multivariate pattern analysis (MVPA) techniques, achieving a better classification accuracy. However, there are limitations to this approach that stem from small sample sizes, scanner variability, and the absence of a comprehensive independent data set. By computing the Hurst exponents of resting-state networks, researchers examined their long-term memory for distinguishing depressive patients from HCs (17). Scale-free dynamics of depression-related brain activity were seen as describing the long-term memory of resting-state networks. An SVM-based classifier was used to test the data with a leave-one-out cross-validation (Loocv) method. Others studied the effects of MDD and schizophrenia on whole brain R-fMRI using SVM based MVPA in Yu et al. (18), Lois and Wessa (19), Zhu et al. (20), and Li et al. (21). Again, the dimensionality reduction technique relied on the Loocv strategy due to the small sample size. However, it is essential to evaluate the classification performance of these perspectives using a larger sample of subjects. Others showed that whole-brain R-fMRI connectivity may effectively predict antidepressant medication status in people with serious MDD (22). Medication-naive patients were distinguished from controls by the use of a trained linear SVM classifier based on MVPA technique. A different MVPA strategy based on linear, radial basis function (RBF)-SVM classification with the elastic net feature selection technique could accurately distinguished MDD patients from control subjects (23). The hyper-networks in this study were built using an elastic net and the group lasso technique. Hyper-edge, brain area, and average metric analyses suggested that the hyper-networks built with elastic net and group lasso differed structurally (24). Further, functional connection density measures derived by R-fMRI have shown to be successful to determine the relationship between the changes in resting-state activities and the responses to electroconvulsive therapy in 23 patients with MDD (25). The neural indices that were discovered as classification criteria were entered into linear SVM based MVPA, which was then used to categorize MDD patients. In another study, the identification of MDD among subjects, was explored *via* different static and dynamic connection metrics retrieved from R-fMRI (26). In this study a feature vector for classification was built by combining features from static and dynamic techniques. To determine the final predictor performance, a Loocv procedure was implemented. Differential sub-graph entropy and dynamic connectivity characteristics have been used in an SVM classifier to distinguish between people with MDD and HCs. A sliding-window approach was implemented to determine functional connectivity in the context of dynamic processes (27). The dynamic functional connectivity matrices were then employed as features in a non-linear SVM model to differentiate MDD patients from controls. Analysis techniques based on the minimum spanning tree need the computation of measurable qualities and the selection of these attributes as features in the classification. Using two feature types to measure two aspects of the network, a multi-kernel SVM classification method (28) allows the use of both brain region features and subgraph features. All studies discussed above made use of an approach that is based on linear SVM, multi-kernel SVM and RBF-SVM to differentiate between MDD patients and HCs.

Some other studies have used different ML methods, such as regression and neural networks, to differentiate between individuals with MDD and HCs. Using partial least squares regression on R-fMRI data, researchers developed a low-dimensional representation that links symptoms to brain activity and predicts clinical measures (29). R-fMRI connectivity in another study was calculated by employing the automated anatomical labeling layout with a partial correlation method (30). The calculations of the metrics and the classification analysis were performed in the frame of neural network. However, the efficacy and sorting of selected features, as well as sample size, kind of classifiers, and distribution of data, all have a role in determining the appropriate amount of features. For example, when developing a classifier for melancholy MDD (31), it has been shown that is it crucial to identify vitally relevant functional connections. It is for such cases recommended to use logistic regression to evaluate the uniformity vs. heterogeneity connectivity hypotheses.

The concept of deep learning has recently received significant attention. Notably, graph-based techniques, such as GNN, have been used to investigate detailed node pair in imaging/nonimaging characteristics among participants, with the goal of identifying significant phenotypes for clinical identification. Successfully applying a whole-brain data-driven approach with R-fMRI, confirmed the use of effective connectivity for MDD detection by calculating its measures *via* a group sparse representation and a structured equation modeling approach (32). Successful integration of effective connectivity and nonimaging phenotypic information allowed the use of spectral graph convolutional networks (GCN) based on a population graph to differentiate drug-naive MDD patients from HCs. Using functional connectivity as a characteristic, Ktena et al. (33) trained a spectral GCN with subjects as nodes. The spectral GCN was used to diagnose the problem by grouping the nodes into their respective categories. Others used a mutual multi-scale triplet GCN (34) for the purpose of analyzing static FC and structural connectivity with the intention of identifying brain disorders. Further a spatio-temporal GCN framework was created to train discriminative features from FC measures for the automated identification and treatment response prediction of MDD (35). The GCN model was developed to every participant's whole-brain functional network in order to differentiate MDD patients from HCs, recognize the most important regions making a contribution to classifying, and investigate the association between structural features of salient regions and clinical features (36).

In this research, our main aim is to employ an ensemble-based GNN framework to perform the primary classification analysis between MDD and HCs as well as subgroup analysis between first-episode and recurrent (REC). Instead of using a single unified GNN model to learn representations for all of the nodes in a large graph, it is better to use ensemble learning methods (37) to improve classification performance. With ensemble learning, many fundamental classifiers are combined to boost the predictive power of the model. Therefore, to enhance the efficiency of scalable GNN, we propose a GNN-based ensemble model that creates customized models.

### 1.1. Aims

This study provides a methodology for MDD analysis and classification using brain functional networks derived from R-fMRI data. Since the brain is a complex network system, the present study analyses the R-fMRI as the whole brain functional structure rather than individual FCs. In this research, our main aim is to employ an ensemble- based GNN framework to perform the primary classification. Our main scientific contributions are as follows:

- Developing a robust ensemble-based GNN model that takes R-fMRI data for the detection of MDD. A created novel ensemble model is used for identifying individuals with MDD from HCs as well as to perform analysis between two sub groups of MDD patients, namely first episode drug nive (FEDN) and recurrent (REC) MDD patients.
- GCN, GAT, and GraphSAGE models are created as the base line models for the ensemble model, for improving classification accuracy. A developed ensemble model is trained using individual whole-brain functional networks.
- Methods of upsampling and downsampling are employed to achieve balanced sample size. A 10-fold enumeration is used to refine the classification process. Empirical investigations with a large sample size showed that our model is more accurate and beneficial for classification of MDD compared to other models that are currently available.

## 2. Materials and methods

### 2.1. Subjects

R-fMRI data from the REST-meta-MDD collaboration (6), which contained 25 datasets totaling 2428 persons (1300 MDD patients and 1128 HCs from 17 hospitals), was used in the present study. There have been 562 patients with MDD who were experiencing their first episode of the disorder, as well as 282 patients with MDD who have been experiencing recurring episodes of the disorder. According to a previous publication on the dataset (6), we used criteria such as missing data, low-quality spatial normalization, insufficient coverage, noticeable head movement, and sites with fewer than 10 subjects to exclude. This was produced in a sample of 821 people with MDD and 765 HCs from 16 different sites. Drug consumption data was submitted by 527 patients; 219 of these individuals are currently using first episode MDD patients without medication treatment was defined as FEDN, and REC is the MDD patients with recurrent episode regardless of medication status. Two research groups (Sites 5 and 13) contributed data on 117 FEDN patients and 72 patients with REC MDD, five research groups (Sites 4, 5, 9, 13, and 16) contributed data on 227 FEDN patients and 388 HCs, and six research groups (Sites 3, 5, 7, 12, 13, and 14) contributed statistics on 189 patients with REC and 423 HCs. The studies involving human participants data were reviewed and approved by the Institutional Review Board of Kunsan National University.

### 2.2. Preprocessing

Data from R-fMRI and structural MRI were acquired and the DPARSF toolkit (38) was used to perform preprocessing procedure. Slice timing correction, head motion correction, normalization, and the elimination of confounds were the main preprocessing procedures. Dosenbach's atlas was used as a reference point during the process of segmenting the entire brain into 160 distinct regions of interest (ROI) (39). The voxel-level BOLD values were extracted and averaged across all ROIs. The Pearson correlation coefficient of the related time series was used to assess FC between each pair of ROIs. Finally, the correlation estimates were transformed using Fisher's z-transform to generate FC matrix in the range of 160 × 160 for each subject (40).

### 2.3. Methods

**Figure 2:**
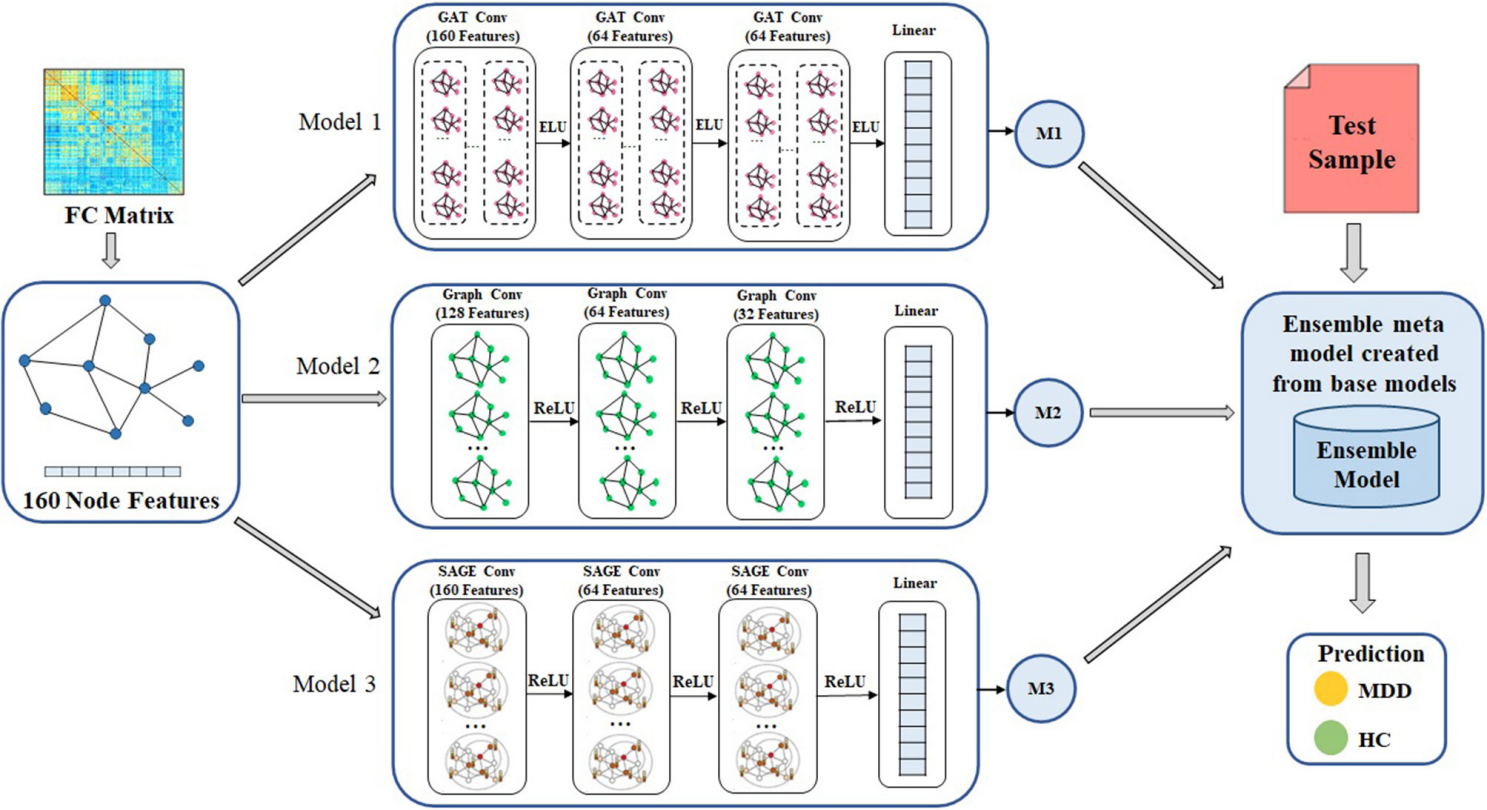
Overall framework of the proposed method for MDD classification. Ensemble model is designed to create meta model from the out features of each base models. GAT, graph attention network; GCN, graph convolutional network; SAGE, GraphSAGE technique, ReLU, Rectified Linear Unit; ELU, Exponential Linear Unit, MDD, major depressive disorder; HC, healthy controls.

The overall process of the GNN-based ensemble model is shown in Figure 2. The FC matrix the whole brain is initially depicted as a weighted undirected graph *G*(*N, E*), where *N* and *E* are collections of nodes and edges. Nodes are the 160 brain regions identified by the ROIs, and their characteristics are the matrix representation of the functional connection between them. The link between nodes are represented by a weighted adjacency matrix (A). Each node is linked to its nearest neighbors using a k-nearest neighbors (KNN) technique to establish edges (36). In order to construct the GNN-based ensemble model, the GCN, GAT, and SAGE models are used as the base models. The core operation of an ensemble model is to combine out features of base models and apply the softmax activation function to translate final scalers into predicted probabilities of the each class.

#### 2.3.1. Base models

**Figure 3:**
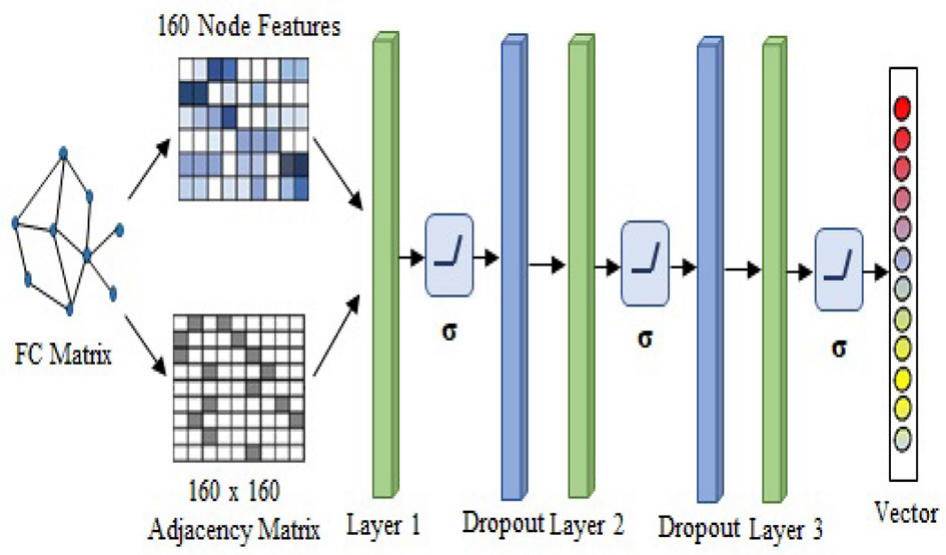
Structure of the base model.

First, the FC matrix was represented as a graph structure, along with an adjacency matrix and node characteristics. The GAT model uses a graph attention layer to learn the node representation, followed by an attention pooling layer and a classification layer to retrieve the node representation and perform the task of learning. We began by stacking three GAT layers with exponential linear unit (eLU) activation functions, then moved on to a global mean pooling layer, then a dropout layer, and finally fed that information into a classification layer. The head size is 8 and the dropout rate is 0.5 in the GAT model. Input layer, graph convolutional hidden layer, fully connected layer, and global average pooling layer are the components that make up GCN model. After each hidden layer, there is a rectified linear unit (ReLU) activation function. The dropout rate is 0.3 in the GCN model. We also use GraphSAGE with three layers (*K* = 3) as a base model. The dimensions of the node embeddings are the same as the size of the hidden units that are used in each GraphSAGE layer, which is 64. The ReLU activation function and a dropout rate of 0.3 are used in each of the GraphSAGE layers. Also, Every model has a weight decay value of 5 x 10^−4^ and a learning rate of 0.01. Each model in GNN, such as GCN, GAT, and GraphSAGE, is trained with the same set of node features, edge features, weights, and learning rate. Figure 3 illustrates the processes involved in creating a base model.

#### 2.3.2. Ensemble model

**Figure 4:**
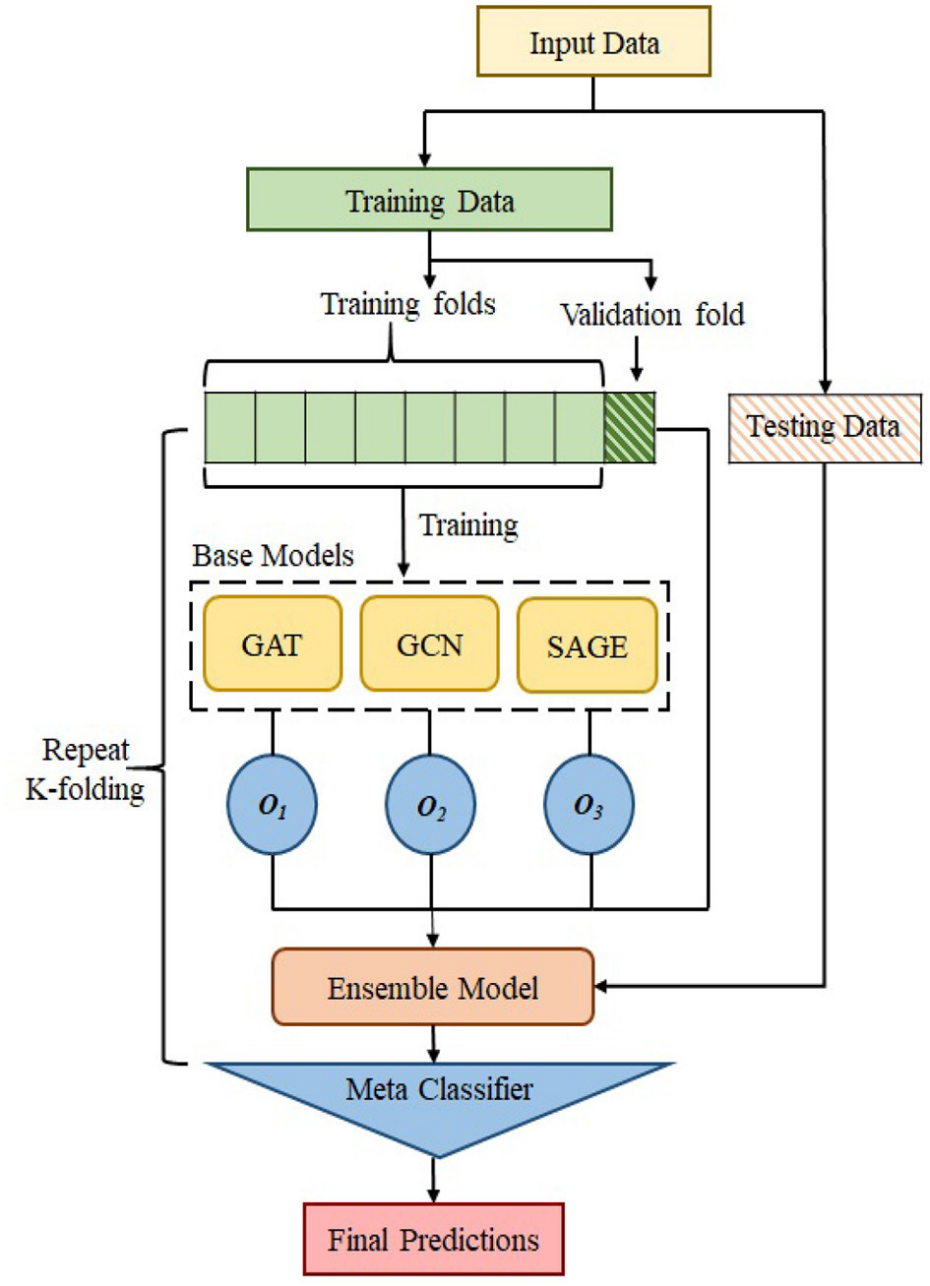
Flowchart to show the process of ensemble model.

We use the ensemble-based GNN model in order to determine the essential features that contribute to the prediction of MDD. The suggested ensemble-GNN procedure is depicted in the flowchart described in Figure 4. We construct a GNN-based ensemble model using GCN, GAT, and GraphSAGE as building blocks. Both the node features and the adjacency matrix are fed into the base models, and the features' identities are then extracted from the respective models. Each model's predicted output features are fed into the ensemble model. Then, for class prediction, we add fully connected layers with a softmax activation function. The cross-entropy loss function is put into effect to this extent. The adam optimizer is used to find the optimal values for each of the model's parameters.

### 2.4. MDD identification and evaluation

MDD classification employs ensemble-based GNN supervised learning classifiers, and this classification makes it simple to compare the efficacy of various machine learning strategies for processing fMRI data. Both training and testing are required for supervised learning classification. With the help of the samples' class labels, the classifier identifies a decision boundary that divides the input space during the training phase. Once the decision function has been calculated using the training set, it may be applied to unseen testing data to infer the corresponding class label. In order to reduce the overfitting problem and to offer a reliable and generalizable classification performance evaluation, the effectiveness of the classification framework is evaluated using the 10-fold cross validation scheme. In order to balance the sample size, oversampling is accomplished by copying data from minority classes, whereas undersampling is carried out by selecting data from majority classes. The performance of the classification system is measured and analyzed based on its accuracy (ACC), specificity (SPE), sensitivity (SEN), and area under the curve (AUC). The diagnostic accuracy of a classifier can be measured with the use of receiver operating characteristic, which is a curve that is generated by graphing the true positive rate against the false positive rate. The process of determining classification ACC, SPE, and SEN is denoted as,


(1) $$\begin{array}{l} ACC=\frac{TP+TN}{TP+FP+FN+TN} \end{array}$$

(2) $$\begin{array}{l} SPE=\frac{TN}{TN+FP} \end{array}$$

(3) $$\begin{array}{l} SEN=\frac{TP}{TP+FN} \end{array}$$

In this case, TP indicates a successful classification of positive samples, TN indicates a successful classification of negative samples, and FP indicates incorrect classification of negative samples as positive, and FN indicates incorrect negative classification.

## 3. Results

In this section, we validate the efficiency of the suggested MDD identification approach by analyzing the following scenarios: (a) using FC as features; (b) using GCN, GAT, and GraphSAGE as base learners; (c) using an ensemble classification model. We used a 10-fold cross-validation, and in that cross-training, we split the samples of all MDD and HCs into 10 groups. Each time the method is modified, one unit is chosen as the testing dataset for assessing the performance of the model, while the remaining 9 units are used as the training dataset. By stratifying the 10-fold cross-validation, we are able to keep the percentage of samples from each class in every fold equal across the entire sample. In this case, the samples are not balanced, so in order to create samples that are balanced, random upsampling is performed on the majority classes, and random downsampling is performed on the minority classes. For the primary analysis, there were a total of 1,586 participants included in our method (821 patients with MDD and 765 HCs). Based on the clinical data for the patients who are included, 243 were FEDN patients, and 203 are REC patients.

### 3.1. MDD vs. HC classification

**Figure 5:**
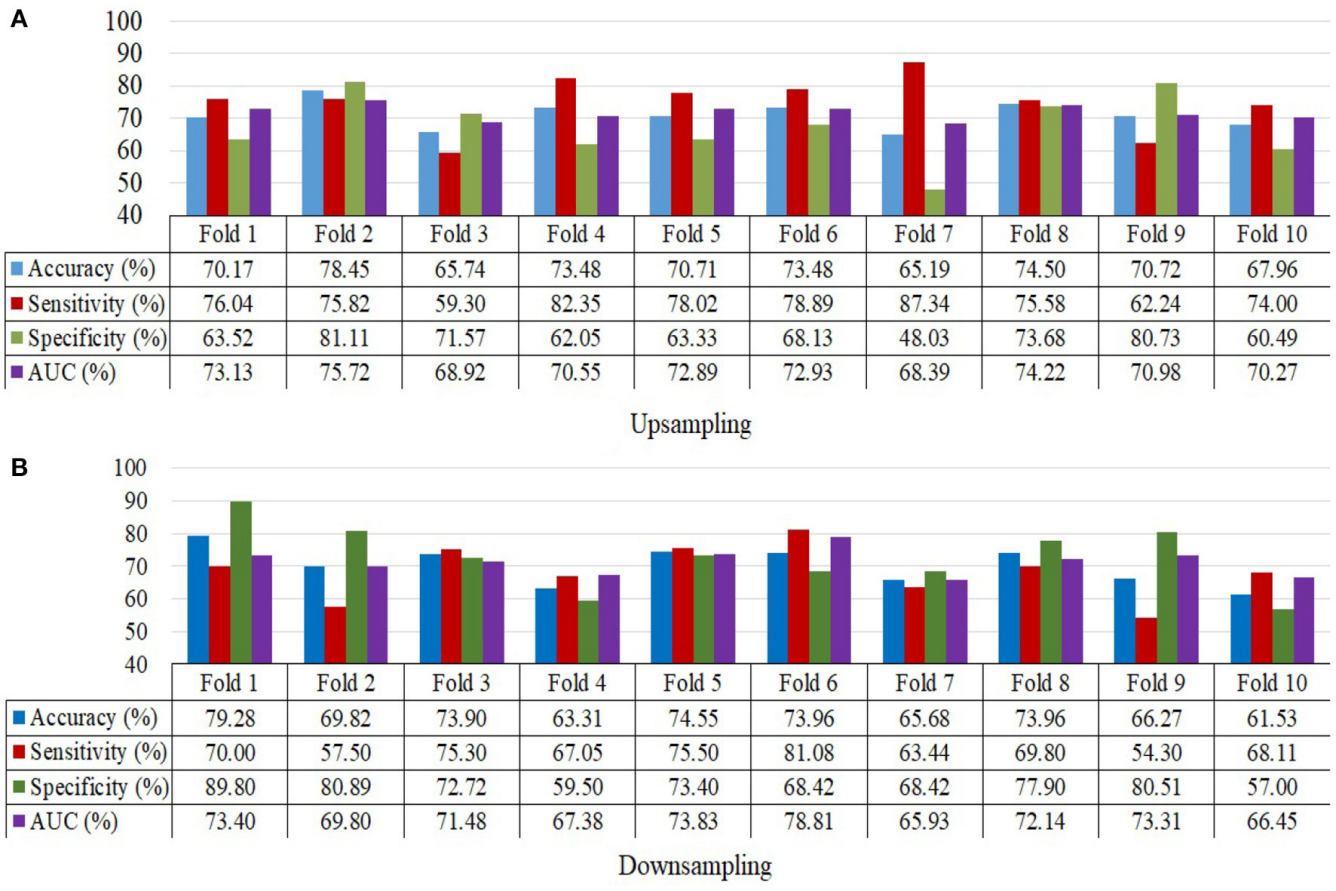
10-Fold cross validation for MDD and HC samples depicting accuracy, sensitivity, specificity and the area under the curve. **(A)** Upsampling and **(B)** Downsampling.

The ensemble model attained an accuracy of 71.8% for upsampling and 70.4% for downsampling when it came to classifying MDD and HC. When using upsampling, the ensemble model achieves an AUC of 76.53%, while using downsampling, it achieves an AUC of 71.27%. Specificity and sensitivity values for upsampling are 74.96 and 68.23%, respectively, whereas the values for downsampling are 67.27 and 72.88%. The findings for upsampling and downsampling based on a 10-fold cross validation for MDD and HC classification are given in Figure 5.

### 3.2. FEDN vs. HC classification

FEDN could be distinguished from HCs with a classification accuracy of 88.93% for upsampling and 64.17% for downsampling. Classification of FEDN patients with HC achieved an upsampling AUC of 85.75% and a downsampling AUC of 62.25%. Upsampling has a specificity and sensitivity of 89% and 85.79%, whereas downsampling has a specificity and sensitivity of 60.31 and 61.36%. The findings from classifying FEDN and HCs are shown in Table 1.

**Table 1:** Ensemble model performance for FEDN vs. HC classification.

**Ensemble Model - FEDN vs. HC**				
**Sampling**	**ACC**	**SEN**	**SPE**	**AUC**
Upsampling	0.8893	0.8900	0.8597	0.8584
Downsampling	0.6417	0.6031	0.6138	0.6225

### 3.3. REC vs. HC classification

Classification accuracy for upsampling REC with HC is 91.6%, whereas classification accuracy for downsampling REC patients with HC is 68.78%. Using upsampling, we are able to classify REC patients as distinct from HC with an AUC of 88.24%, while using downsampling, we are only able to reach an AUC of 67.11 %. The respective results for specificity and sensitivity while upsampling are 93.15 and 87.20%, while they are 60.96 and 66.92% when downsampling. Table 2 describes the results produced from the classification of REC and HCs.

**Table 2:** Ensemble model performance for REC vs. HC classification.

**Ensemble Model - REC vs. HC**				
**Sampling**	**ACC**	**SEN**	**SPE**	**AUC**
Upsampling	0.9160	0.9315	0.8720	0.8824
Downsampling	0.6878	0.6096	0.6692	0.6711

### 3.4. FEDN vs. REC classification

The FEDN and the REC are discriminated from each other by the use of a subgroup analysis. There is a 77.78% accuracy rate when upsampling FEDN against REC and a 71.96% rate when downsampling. When we compared the existing approach (36) to the subgroup analysis, we found that the classification performance for the characterization of recurring patients was greater than that of FEDN. Using upsampling, we achieved an AUC of 75.19%, whereas using downsampling, we achieved an AUC of 71.77%. Upsampling yields results of 72.81 and 81.91% for specificity and sensitivity, whereas downsampling yields results of 71.52 and 71.56%. The outcomes of the FEDN and REC classifications are shown in Table 3.

**Table 3:** Ensemble model performance under FEDN vs. REC classification.

**Ensemble Model - FEDN vs. REC**				
**Sampling**	**ACC**	**SEN**	**SPE**	**AUC**
Upsampling	0.7778	0.7281	0.8191	0.7519
Downsampling	0.7196	0.7150	0.7152	0.7177

## 4. Discussion

**Figure 6:**
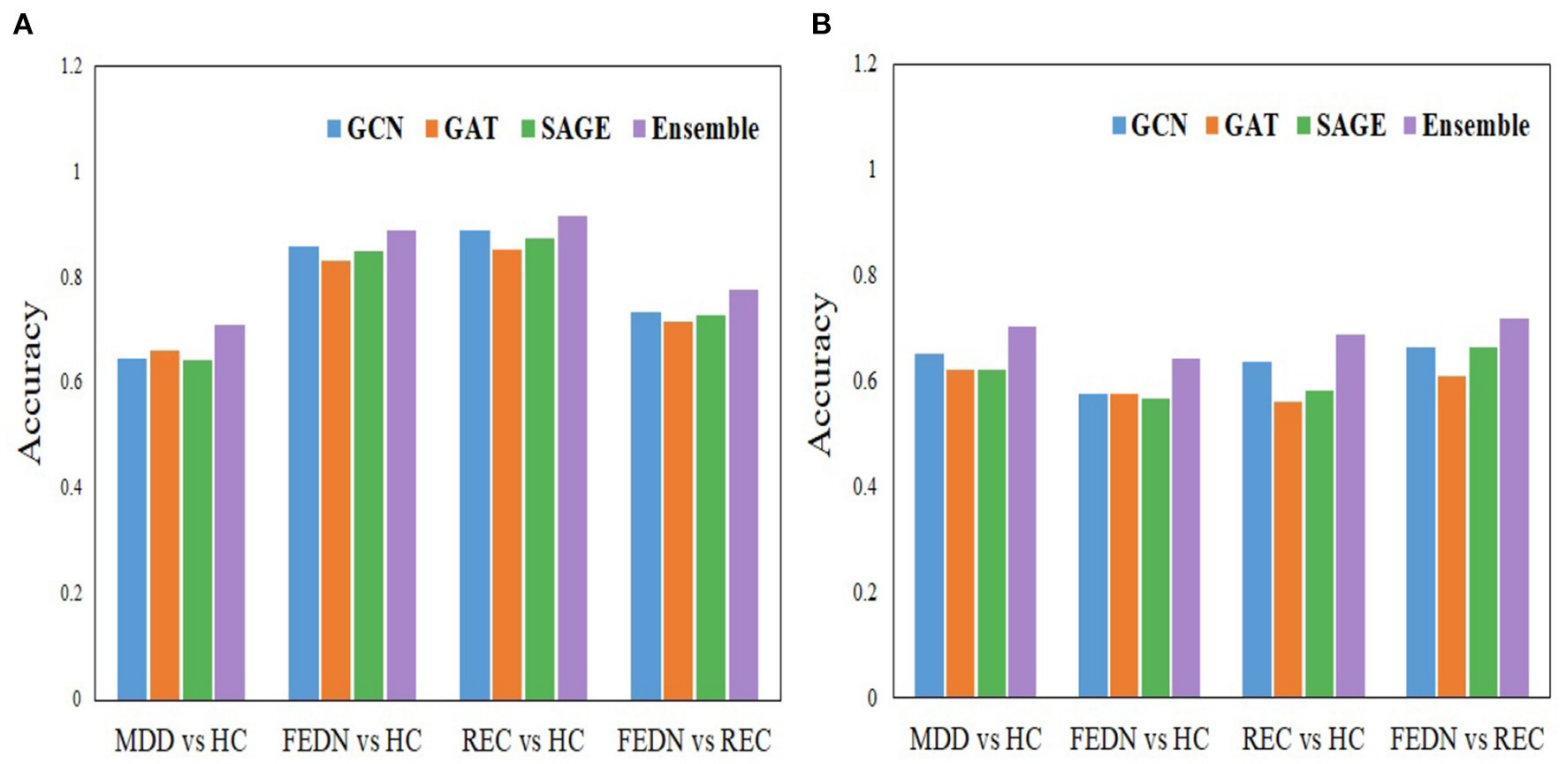
Comparison analysis between the individual model and the ensemble model. **(A)** Upsampling and **(B)** Downsampling.

We proposed an ensemble-based GNN method for automatic MDD identification using whole brain functional network features. Using a large open source dataset, the current study employed an ensemble based GNN to classify MDD as well as to classify FEDN with REC, and the resulting upsampling classification performance outperformed typical machine learning approaches by around, 71.18% and 77.78%, respectively. In the analysis process, initially, separate base models are created, and then the classification performance of each model is examined. Models such as GCN, GAT, and GraphSAGE are employed as base line models. The GAT approach is utilized during the training of the model, which resulted in an upsampling accuracy of 66.24% when comparing MDD to HC and 71.67% when comparing FEDN to REC. The GCN technique is also separately applied during the training process of the model, which led to an upsampling accuracy of 64.72% while correlating MDD to HC and 73.58% while comparing FEDN to REC. Also, the GraphSAGE model alone was employed when training the model, which produced in an upsampling accuracy of 64.47 % for MDD among HC classification and 72.78% for FEDN with REC classification. In addition to this, we used an all-individual model to analyze subgroups such as FEDN with HC and REC with HC. The results of the individual base models such as GCN, GAT, and GraphSAGE are listed in Table 4. The GNN-based ensemble model is developed to improve the classification accuracy of primary analysis as well as subgroup analysis in analyzing MDD. Already, the base models are trained independently, and while some findings indicate that GCN produces better results, other findings show that GAT or GraphSAGE produces better outcomes. That indicates that no one model achieves better results for all classes. Because of this, a combined model is developed to produce more accurate results for the whole sample. Figure 6 displays the results of a comparison between the base model and the ensemble model.

**Table 4:** Base model performance under different group analysis.

**Model**	**Upsampling**				**Downsampling**			
	**ACC**	**SEN**	**SPE**	**AUC**	**ACC**	**SEN**	**SPE**	**AUC**
**MDD vs. HC**								
GCN	0.6472	0.6171	0.6537	0.7122	0.6519	0.5991	0.7041	0.7089
GAT	0.6624	0.6619	0.6667	0.6734	0.6219	0.6176	0.6270	0.6640
SAGE	0.6447	0.5968	0.6715	0.7220	0.6213	0.6713	0.5705	0.6859
**FEDN vs. HC**								
GCN	0.8593	0.8599	0.8588	0.8543	0.5759	0.7263	0.4188	0.6185
GAT	0.8320	0.8299	0.7929	0.8387	0.5759	0.6446	0.4862	0.6200
SAGE	0.8520	0.8780	0.7986	0.8692	0.5667	0.5304	0.5994	0.6211
**REC vs. HC**								
GCN	0.8913	0.9397	0.8728	0.9051	0.6363	0.5936	0.6824	0.6625
GAT	0.8527	0.8846	0.7929	0.8534	0.5609	0.5763	0.5828	0.6653
SAGE	0.8746	0.8889	0.8627	0.8946	0.5826	0.5964	0.5705	0.6035
**FEDN vs. REC**								
GCN	0.7358	0.7069	0.6822	0.7653	0.6651	0.6562	0.6776	0.7101
GAT	0.7167	0.6876	0.7267	0.7113	0.6109	0.6420	0.5945	0.7016
SAGE	0.7278	0.6608	0.8000	0.7539	0.6652	0.7138	0.6166	0.7126

In the majority of the earlier investigations, the ML algorithm was employed to differentiate between all MDD and HCs (20, 41–43). To identify brain disorders, researchers have created a number of deep learning techniques, including BrainNetCNN (44) and discriminative/generative long short-term memory (45). However, the sample size that they employed for the investigation was comparatively small. The use of GNN to distinguish between MDD and HCs has been proven effective in a small number of studies. Some previous research (32, 34, 46) has shown that the graph convolution technique can be used to distinguish disorders in patients with HC. This is in contrast to others, who employed MVPA of static or dynamic functional connectivity in the brain network (47, 48), which neglected topological elements that could provide key clues for diagnosis. The ensemble-GNN algorithm combines the results of numerous GNN classifiers into a single model in order to minimize the impact of overfitting. In addition, by using ensemble-GNN, the deviation caused by a single classifier can be reduced, resulting in improved reliability. When applied to imbalanced datasets with the same number of learning epochs, ensemble-GNN obtains a higher classification accuracy than a single classifier does. We took primary analyses into consideration such as all MDD with HCs as well as subgroup analyses including FEDN among HCs, REC with HCs, and FEDN along with REC. In addition, We also analyzed the model using the Craddock and Automated Anatomical Labeling (AAL) atlases. Our ensemble GNN model achieves an accuracy of 74.75% for the AAL atlas and 73.37% for the Craddock atlas when classifying MDD vs. HC upsampling data. Tables 2, 4 of the Supplementary material contain the primary and all of the sub class analysis results for the AAL and Craddock atlas, respectively.

## 5. Limitations

Some limitations should be taken into account when evaluating the present findings. We were not able to reach the classification performance in the case of MDD with HC categorization when compared with the previous approach (36). However, our methods achieve higher performance in subgroup classification. In order to solve the problem of imbalanced class representation, we tried both random sampling. Upsampling gives better results, but it can add a redundant samples to the model, which slows down training and vulnerable to overfitting. In our approach, the overfitting problem is reduced by utilizing cross-validation; however, training speed is not taken into account. Also, with respect to the population, most MDD was females which may have a confounding effect on MDD classification. However, it should be noted that the same GNN model structures could also successfully classify between MDD subgroups, FEDN vs. REC, implying that MDD classification is not entirely dependent on the sex effect. Further studies that aim to test this effect requires a much larger sample size for controlling sex or adequate matching.

## 6. Conclusion

In this study, we effectively created an ensemble model based on GNN for classifying MDD by utilizing R-fMRI data. In particular, we investigated a sub-group analysis between FEDN with REC. The proposed model that employs whole brain functional connectivity classifies MDD patients and healthy individuals with high accuracy. In order to improve the overall performance of the ensemble model, we used three different GNN base models under 10-fold cross-validation. Based on large dataset and a number of different of validation techniques, an ensemble model could classify MDD and HCs with a feasible accuracy of 71.18% for upsampling and 70.14% for downsampling. When compared to earlier approaches, the findings that these methods yield in the subgroup analysis are higher. This method achieves an accuracy of 77.78% for upsampling and 71.96% for downsampling when applied to an analysis of FEDN and REC. The findings of this validation suggest that our model produces a feasible application to assist healthcare professionals in identifying MDD.

## Data availability statement

The original contributions presented in the study are included in the article/Supplementary material, further inquiries can be directed to the corresponding author.

## Ethics statement

The studies involving human participants were reviewed and approved by the Institutional Review Board of Kunsan National University. Written informed consent from the was not required to participate in this study in accordance with the national legislation and the institutional requirements.

## Author contributions

SV, MV, LW, SK, ML, UH, I-HR, and H-GJ contributed to conception and implementation of the study. SV wrote the first draft. All authors contributed to review, editing, and approved the submitted version.
