# Supplementary material for: Ensemble graph neural network model for classification of major depressive disorder using whole-brain functional connectivity
Source: Front Psychiatry. 2023 Mar 23;14:1125339. doi: 10.3389/fpsyt.2023.1125339 (PMC10077869; doi:10.3389/fpsyt.2023.1125339)
Supplement: Supplementary file 1 [file Data_Sheet_1.PDF]

Supplementary Material

Correspondence*:
Han-Gue Jo
hgjo@kunsan.ac.kr

1 GRAPH STRUCTURE

An undirected weighted graph, denoted by the expression $G = (N, E, W)$, has a set of nodes that ranges from 1 to n (160), a set of edges that equals $E \in NxN$, and an adjacency matrix W, where the notation $(j, i) \in E$ signifies an edge that connects node j to node i. Adjacency matrix W represents the connections between nodes in N, where the ith row and j^{th} column of W represent the connection between the ith and j^{th} nodes. Consider, h_i^0 is the initial feature vector for node i.

2 GRAPH ATTENTION NETWORK

The attention mechanism is embedded into the various stages of the graph attention network (GAT) propagation mechanism. A graph attention layer receives a set of node features as input, $h = \overrightarrow{h_1}, \overrightarrow{h_2}...\overrightarrow{h_N}$, where N is the total number of nodes in the graph. The attention layer generates new node features, which may have different dimensions than the input features. The nodes then conduct self-attention in order to calculate attention coefficients.

$$e_{ij} = a(W\overrightarrow{h_i}, W\overrightarrow{h_j}) \tag{1}$$

where, W is a common linear weight matrix for all nodes and e_{ij} represents the significance of node j's attributes to node i. In most cases, only first-order neighbors are taken into account for determining attention coefficients. The activation function is used to standardize the attention coefficients for all values of j.

$$a_{ij} = \frac{exp(e_{ij})}{\sum_{k \in N} exp(e_{ik})} \tag{2}$$

While applying a nonlinearity, the normalized attention coefficients are used to update the appropriate features in order to calculate an output value for each node. When K distinct attention heads are used to independently compute features, the results can be combined (4). The notation for a node created by multiple heads of attention is:

$$h_i^{'} = \sigma\left(\frac{1}{K}\sum_{k=1}\sum_{j \in N_i} a_{ij}^k W^k h_j\right) \tag{3}$$

Prior to being fed into a classification layer, a graph's final representation is always aggregated using an attention pooling layer.

3 GRAPH CONVOLUTIONAL NETWORK

Graph convolution network (GCN) mainly focuses on the spectral graph convolution, which can perform the convolution operation on an irregular graph. L is the normalized lablacian of the graph (1), represented

as,

$$L = I_n - D^{\frac{-1}{2}} W D^{\frac{-1}{2}} \tag{4}$$

where, I be the identity matrix and D is the diagonal matrix of node degree,$D_{ii} = \sum_j A_{i,j}$. The laplacian matrix of the normalized graph has the property of being real symmetric positive semidefinite. Because of this, the normalized laplacian matrix can be factored as, $L = U \wedge U^T$. where, U is the matrix of eigenvectors in increasing eigenvalue order, and $\wedge$ is the diagonal matrix of eigenvalues. By considering the node attribute and the graph convolution filter, spatial graph convolution can be represented as a spectral multiplication:

$$g_\theta(L)x = g_\theta(U \wedge U^T)x = U g_\theta(\wedge) U^T x \tag{5}$$

Polynomial parametrization is applied to reduce computing costs and localizes filters. ChebNet (5) approximates filter g_θ by Chebyshev polynomials of diagonal eigenvalues, $g_\theta = \sum_{i=0}^{K} \theta_i T_i(\tilde{\wedge})$ where $\tilde{\wedge} = 2 \wedge / \lambda_{max} - I_n$ and $\wedge$ values are in between [-1, +1]. Iteratively, the Chebyshev polynomials can be represented as $T_i(x) = 2XT_{i-1}(x)T_{i-2}(x)$ with $T_0(x) = 1$ and $T_1(x) = x$. As a consequence of this, the convolution of a graph signal x with the specified filter g_θ is,

$$x * g_\theta = U \sum_{i=1}^{K} \theta_i T_i \tilde{\wedge} U^T x \tag{6}$$

where, $\tilde{L} = \frac{2L}{max} - I_n$. Since, $T_i(\tilde{L}) = UT_i(\tilde{\wedge})U^T$ that can be determined using induction on i ChebNet has the expression,

$$x * g_\theta = U \sum_{i=0}^{K} \theta_i T_i(\tilde{L}) x \tag{7}$$

In consequence, the following expression describes the output of the nth graph convolutional layer for a given sample:

$$y_i^n = \sum_{j=1}^{F_{in}} g_{\theta}{}_j^n T_i(L) x_{i,j}^n \tag{8}$$

F_{in} be the input filter and $x_{i,j}^n$ is the input feature map for sample i at n^{th} layer. $g_{\theta}{}_j^n$ represents the learnable parameters of K-order chebyshev polynomials (3).

4 GRAPHSAGE NETWORK

First, each node N gives a representation of the nodes belonging to its immediate neighborhood as a vector$h_{I(N)}^k$. This summation step depends on the representation made in the previous iteration step $(k-1)$ (2).

$$h_{I(N)}^k = AGGREGATE_k(h_u^{k-1}, \forall u \in I(N)) \tag{9}$$

The distance (k) determines how many neighboring nodes are sampled, and each node's representation is denoted by its hash value h_k. After combining the neighboring feature vectors, GraphSAGE combines the node's current representation,h_N^{k-1} with the combined neighborhood vector, $h_{I(N)}^{k-1}$ and sends this combined vector through a fully connected layer with a nonlinear activation function σ.

$$h_N^k = \sigma(W^k.CONCAT(h_N^{k-1}, h_{I(N)}^k)) \tag{10}$$

For convenience, we denote the final representations output at depth K as, $Z_n = h_n^k \forall n \in N$

5 ENSEMBLE SETUP

Nodes are the 160 brain regions that have been identified by the ROIs, and their characteristics are the matrix representation of the functional connection between them. For the structure of our ensemble model, we used three hidden layers with 128, 64, and 32 filters in each layer for GCN and 64 filters for all layers in GAT and GraphSAGE, respectively. Cross entropy loss function and the Adam optimizer were used to measure the loss and update the model's parameters. To reduce the risk of overfitting, the second and third layers had a dropout rate of 0.3 for GCN, GraphSAGE, and 0.5 for GAT, and the loss function had a 5x10-4 regularization term. The initial learning rate for Adam optimizer was set to 0.001. The ensemble model should employ the SoftMax activation function. Model training was done for 50 epochs. Pytorch and Scikit-Learn library were used to carry out all training and testing procedures. Table 1 and Table 3 show the upsampling and downsampling classification performances of the individual and combined models, respectively.

FEDN-first-episode drug naïve, REC-recurrent, MDD- major depressive disorder, HC-healthy controls, MDD - 821 samples, HC – 765 samples, FEDN -243 samples, REC-203 samples

Table 1. Classification performance of individual and combined model for Upsampling

Model	FEDN vs. HC	REC vs. HC	MDD vs. HC	FEDN vs. REC
**GCN**	SEN: 0.8599±0.0777<br>SPE: 0.8588±0.0811<br>ACC: 0.8593±0.0494<br>AUC: 0.8543±0.0535	SEN: 0.9397±0.0369<br>SPE: 0.8728±0.0600<br>ACC: 0.8913±0.0278<br>AUC: 0.9051±0.0283	SEN: 0.6171±0.1184<br>SPE: 0.6537±0.0891<br>ACC: 0.6472±0.0460<br>AUC: 0.7122±0.0401	SEN: 0.7069±0.1244<br>SPE: 0.6822±0.1736<br>ACC: 0.7358±0.0816<br>AUC: 0.7653±0.0465
**GAT**	SEN: 0.8299±0.0922<br>SPE: 0.7929±0.0493<br>ACC: 0.8320±0.0509<br>AUC: 0.8387±0.0274	SEN: 0.8846±0.0465<br>SPE: 0.7929±0.0700<br>ACC: 0.8527±0.0457<br>AUC: 0.8534±0.0309	SEN: 0.6619±0.0838<br>SPE: 0.6667±0.0575<br>ACC: 0.6624±0.0352<br>AUC: 0.6734±0.0285	SEN: 0.6876±0.1567<br>SPE: 0.7267±0.0883<br>ACC: 0.7167±0.0927<br>AUC: 0.7113±0.0711
**SAGE**	SEN: 0.8780±0.0770<br>SPE: 0.7986±0.0588<br>ACC: 0.8520±0.0218<br>AUC: 0.8692±0.0208	SEN: 0.8889±0.0336<br>SPE: 0.8627±0.0320<br>ACC: 0.8746±0.0187<br>AUC: 0.8946±0.0285	SEN: 0.5968±0.1463<br>SPE: 0.6715±0.1779<br>ACC: 0.6447±0.0412<br>AUC: 0.7220±0.0223	SEN: 0.6608±0.1155<br>SPE: 0.8000±0.1047<br>ACC: 0.7278±0.0614<br>AUC: 0.7539±0.0636
**GCN +GAT**	SEN: 0.8603±0.0242<br>SPE: 0.8403±0.0540<br>ACC: 0.8515±0.0293<br>AUC: 0.7914±0.0844	SEN: 0.8873±0.0298<br>SPE: 0.8579±0.0411<br>ACC: 0.8722±0.0269<br>AUC: 0.8409±0.0322	SEN: 0.7100±0.0643<br>SPE: 0.6667±0.0858<br>ACC: 0.7033±0.0349<br>AUC: 0.6872±0.0384	SEN: 0.7071±0.1212<br>SPE: 0.7663±0.1249<br>ACC: 0.7541±0.0667<br>AUC: 0.7389±0.0535
**GAT +SAGE**	SEN: 0.8057±0.0255<br>SPE: 0.8332±0.0241<br>ACC: 0.8201±0.0164<br>AUC: 0.8083±0.0182	SEN: 0.8351±0.0556<br>SPE: 0.8481±0.0491<br>ACC: 0.8420±0.0423<br>AUC: 0.8195±0.0345	SEN: 0.7009±0.0649<br>SPE: 0.6143±0.0786<br>ACC: 0.6575±0.0377<br>AUC: 0.6663±0.0345	SEN: 0.7121±0.0712<br>SPE: 0.7687±0.1281<br>ACC: 0.7407±0.0657<br>AUC: 0.7435±0.0807
**GCN+ SAGE**	SEN: 0.8171±0.0338<br>SPE: 0.8615±0.0338<br>ACC: 0.8396±0.0322<br>AUC: 0.8414±0.0429	SEN: 0.8919±0.0387<br>SPE: 0.8670±0.0351<br>ACC: 0.8793±0.0305<br>AUC: 0.8586±0.0192	SEN: 0.7018±0.0589<br>SPE: 0.6444±0.0658<br>ACC: 0.7082±0.0268<br>AUC: 0.7144±0.0229	SEN: 0.7118±0.0550<br>SPE: 0.7649±0.1278<br>ACC: 0.7530±0.0697<br>AUC: 0.7456±0.0685
**GCN+GAT+SAGE Ensemble Model**	SEN: 0.8900±0.0429<br>SPE: 0.8579±0.0451<br>ACC: 0.8728±0.0370<br>AUC: 0.8584±0.0343	SEN: 0.9315±0.0273<br>SPE: 0.8720±0.0422<br>ACC: 0.9024±0.0254<br>AUC: 0.8824±0.0298	SEN: 0.7496±0.0802<br>SPE: 0.6828±0.0952<br>ACC: 0.7140±0.0390<br>AUC: 0.7653±0.0223	SEN: 0.7281±0.0951<br>SPE: 0.8191±0.1189<br>ACC: 0.7778±0.0568<br>AUC: 0.7519±0.0515

Table 2. Classification performance of Ensemble model for AAL and Craddock Atlas - Upsampling
AAL atlas - 116 ROI, Craddock atlas - 200 ROI

Ensemble Model	FEDN vs. HC	REC vs. HC	MDD vs. HC	FEDN vs. REC
**AAL Atlas**	SEN: 0.9004±0.0354<br>SPE: 0.8647±0.0498<br>ACC: 0.8834±0.0261<br>AUC: 0.8757±0.0291	SEN: 0.8978±0.0445<br>SPE: 0.8948±0.0303<br>ACC: 0.8970±0.0116<br>AUC: 0.8632±0.0697	SEN: 0.7307±0.0504<br>SPE: 0.7629±0.0405<br>ACC: 0.7475±0.0185<br>AUC: 0.7386±0.0373	SEN: 0.6821±0.0745<br>SPE: 0.7702±0.0678<br>ACC: 0.7259±0.0412<br>AUC: 0.7161±0.0436
**Craddock Atlas**	SEN: 0.8946±0.0147<br>SPE: 0.8887±0.0446<br>ACC: 0.8917±0.0253<br>AUC: 0.8607±0.0358	SEN: 0.8914±0.0212<br>SPE: 0.8984±0.0232<br>ACC: 0.9021±0.0154<br>AUC: 0.8701±0.0251	SEN: 0.7321±0.0154<br>SPE: 0.7203±0.0134<br>ACC: 0.7337±0.0162<br>AUC: 0.7203±0.0152	SEN: 0.6482±0.1389<br>SPE: 0.7337±0.1267<br>ACC: 0.6815±0.0740<br>AUC: 0.7117±0.0711

Table 3. Classification performance of individual and combined model for Downsampling

Model	FEDN vs. HC	REC vs. HC	MDD vs. HC	FEDN vs. REC
**GCN**	SEN: 0.7263±0.2012<br>SPE: 0.4188±0.2373<br>ACC: 0.5759±0.0582<br>AUC: 0.6185±0.0666	SEN: 0.5936±0.1867<br>SPE: 0.6824±0.1845<br>ACC: 0.6363±0.0841<br>AUC: 0.6025±0.0749	SEN: 0.5991±0.1667<br>SPE: 0.7041±0.1454<br>ACC: 0.6519±0.0606<br>AUC: 0.7089±0.0384	SEN: 0.6562±0.2498<br>SPE: 0.6776±0.2017<br>ACC: 0.6651±0.0648<br>AUC: 0.7177±0.0494
**GAT**	SEN: 0.6446±0.2090<br>SPE: 0.4862±0.1992<br>ACC: 0.5759±0.0757<br>AUC: 0.6200±0.0708	SEN: 0.5763±0.2610<br>SPE: 0.5828±0.2765<br>ACC: 0.5609±0.0806<br>AUC: 0.6653±0.0637	SEN: 0.6176±0.2254<br>SPE: 0.6270±0.2171<br>ACC: 0.6219±0.0785<br>AUC: 0.6640±0.0279	SEN: 0.6420±0.1620<br>SPE: 0.5945±0.1615<br>ACC: 0.6109±0.0950<br>AUC: 0.7016±0.0557
**SAGE**	SEN: 0.5304±0.3092<br>SPE: 0.5994±0.2392<br>ACC: 0.5667±0.0407<br>AUC: 0.6211±0.0601	SEN: 0.5964±0.1819<br>SPE: 0.5705±0.1764<br>ACC: 0.5826±0.0906<br>AUC: 0.6035±0.0878	SEN: 0.6713±0.1227<br>SPE: 0.5705±0.1559<br>ACC: 0.6213±0.0497<br>AUC: 0.6859±0.0265	SEN: 0.7138±0.0793<br>SPE: 0.6166±0.1261<br>ACC: 0.6652±0.0654<br>AUC: 0.7126±0.0671
**GCN +GAT**	SEN: 0.6026±0.1726<br>SPE: 0.5954±0.1465<br>ACC: 0.5963±0.0651<br>AUC: 0.6202±0.0606	SEN: 0.6001±0.1671<br>SPE: 0.5849±0.1507<br>ACC: 0.6326±0.0481<br>AUC: 0.6546±0.0607	SEN: 0.6731±0.0900<br>SPE: 0.7093±0.0829<br>ACC: 0.6907±0.0427<br>AUC: 0.7048±0.0405	SEN: 0.7046±0.1214<br>SPE: 0.6603±0.1001<br>ACC: 0.7087±0.0585<br>AUC: 0.7072±0.0540
**GAT +SAGE**	SEN: 0.6017±0.2294<br>SPE: 0.4845±0.2391<br>ACC: 0.5426±0.0642<br>AUC: 0.6034±0.0752	SEN: 0.6014±0.1918<br>SPE: 0.6319±0.1617<br>ACC: 0.6326±0.0704<br>AUC: 0.6398±0.0772	SEN: 0.6113±0.1239<br>SPE: 0.7020±0.0750<br>ACC: 0.6591±0.0492<br>AUC: 0.6608±0.0377	SEN: 0.6943±0.1153<br>SPE: 0.7032±0.1036<br>ACC: 0.7000±0.0474<br>AUC: 0.7023±0.0645
**GCN+ SAGE**	SEN: 0.6001±0.1602<br>SPE: 0.4829±0.2220<br>ACC: 0.5833±0.1175<br>AUC: 0.6030±0.0887	SEN: 0.6064±0.1246<br>SPE: 0.5943±0.1442<br>ACC: 0.6317±0.0554<br>AUC: 0.6609±0.0751	SEN: 0.6630±0.0888<br>SPE: 0.7119±0.0723<br>ACC: 0.6995±0.0243<br>AUC: 0.6968±0.0431	SEN: 0.7443±0.0947<br>SPE: 0.6736±0.1432<br>ACC: 0.7004±0.0623<br>AUC: 0.6994±0.0889
**GCN+GAT+SAGE Ensemble Model**	SEN: 0.6031±0.1919<br>SPE: 0.6136±0.1702<br>ACC: 0.6019±0.0804<br>AUC: 0.6225±0.0616	SEN: 0.6096±0.1856<br>SPE: 0.6692±0.1673<br>ACC: 0.6370±0.0796<br>AUC: 0.6711±0.1028	SEN: 0.6727±0.0779<br>SPE: 0.7288±0.0953<br>ACC: 0.7024±0.0550<br>AUC: 0.7127±0.0377	SEN: 0.7196±0.1227<br>SPE: 0.7150±0.0694<br>ACC: 0.7174±0.0802<br>AUC: 0.7152±0.0630

Table 4. Classification performance of Ensemble model for AAL and Craddock Atlas - Downsampling
AAL atlas - 116 ROI, Craddock atlas - 200 ROI

Ensemble Model	FEDN vs. HC	REC vs. HC	MDD vs. HC	FEDN vs. REC
**AAL Atlas**	SEN: 0.6804±0.2033<br>SPE: 0.6087±0.2856<br>ACC: 0.6333±0.1007<br>AUC: 0.6500±0.0613	SEN: 0.5440±0.1862<br>SPE: 0.6212±0.1858<br>ACC: 0.5935±0.0695<br>AUC: 0.6017±0.0697	SEN: 0.7493±0.0643<br>SPE: 0.7497±0.0926<br>ACC: 0.7509±0.0520<br>AUC: 0.7602±0.0307	SEN: 0.7584±0.1090<br>SPE: 0.5876±0.2384<br>ACC: 0.6609±0.1057<br>AUC: 0.6871±0.0809
**Craddock Atlas**	SEN: 0.5630±0.1807<br>SPE: 0.7424±0.1258<br>ACC: 0.6537±0.0504<br>AUC: 0.6561±0.0777	SEN: 0.6208±0.2759<br>SPE: 0.5181±0.2875<br>ACC: 0.5435±0.0790<br>AUC: 0.5887±0.0642	SEN: 0.7089±0.1169<br>SPE: 0.7437±0.1117<br>ACC: 0.7254±0.0666<br>AUC: 0.7289±0.0328	SEN: 0.6045±0.2385<br>SPE: 0.7020±0.1265<br>ACC: 0.6587±0.0766<br>AUC: 0.6954±0.0755

REFERENCES

1 .Defferrard, M., Bresson, X., and Vandergheynst, P. (2016). Convolutional neural networks on graphs with fast localized spectral filtering. *Advances in neural information processing systems* 29

2 .Hamilton, W., Ying, Z., and Leskovec, J. (2017). Inductive representation learning on large graphs. *Advances in neural information processing systems* 30

3 .Qin, K., Lei, D., Pinaya, W. H., Pan, N., Li, W., Zhu, Z., et al. (2022). Using graph convolutional network to characterize individuals with major depressive disorder across multiple imaging sites. *EBioMedicine* 78, 103977

4 .Veličković, P., Cucurull, G., Casanova, A., Romero, A., Lio, P., and Bengio, Y. (2017). Graph attention networks. *arXiv preprint arXiv:1710.10903*

5 .Wu, Z., Pan, S., Chen, F., Long, G., Zhang, C., and Philip, S. Y. (2020). A comprehensive survey on graph neural networks. *IEEE transactions on neural networks and learning systems* 32, 4–24
